# Supplementary material for: The causal relationship between the human gut microbiota and pyogenic arthritis: a Mendelian randomization study
Source: Front Cell Infect Microbiol. 2024 Nov 26;14:1452480. doi: 10.3389/fcimb.2024.1452480 (PMC11629706; doi:10.3389/fcimb.2024.1452480)
Supplement: Supplementary file 1 [file DataSheet1.zip › mendelian test/ebi-a-GCST90027577.csv_leave_one_out.pdf]

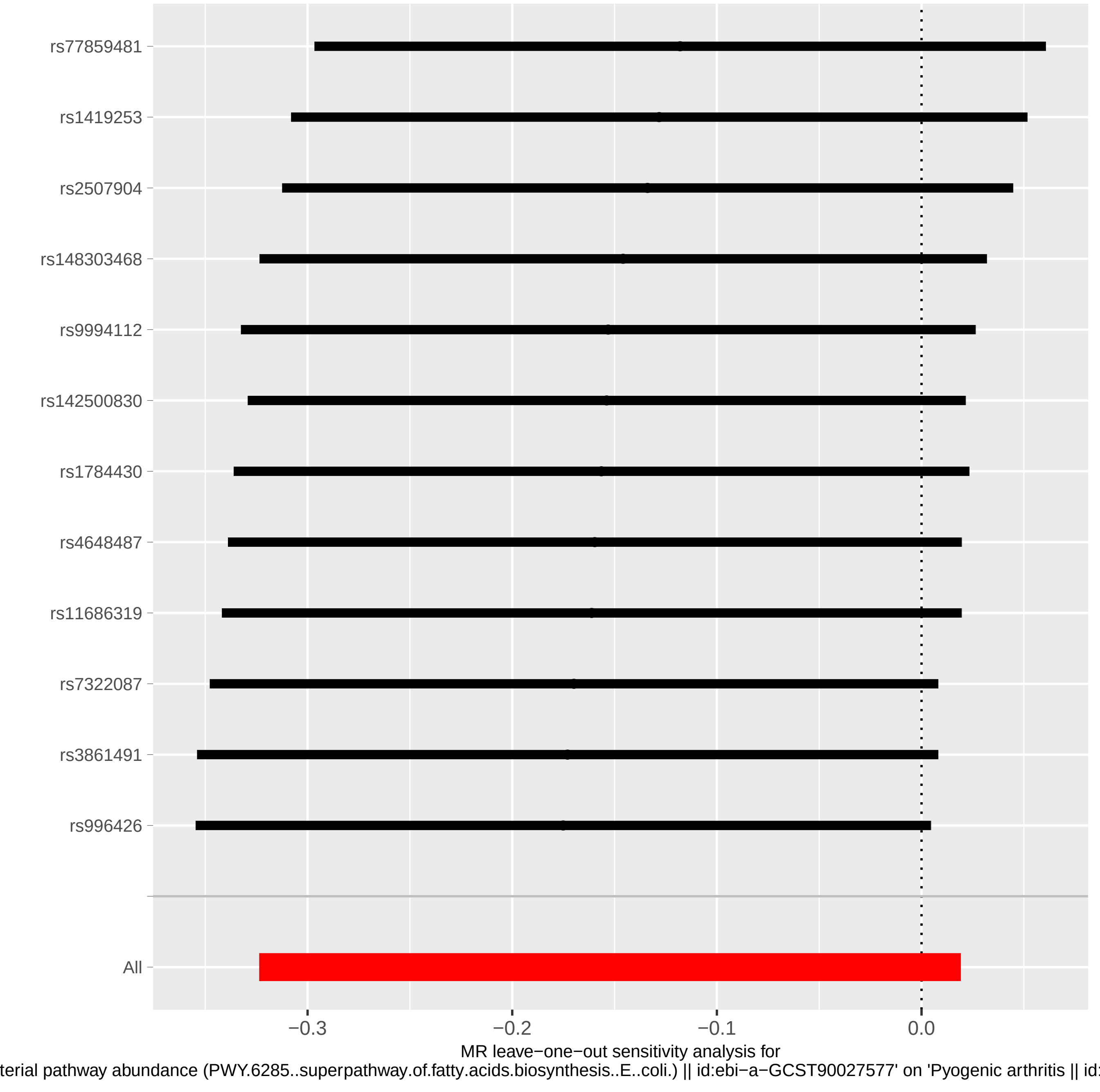
rs77859481
rs1419253
rs2507904
rs148303468
rs9994112
rs142500830
rs1784430
rs4648487
rs11686319
rs7322087
rs3861491
rs996426
All
−0.3
−0.2
−0.1
0.0
MR leave−one−out sensitivity analysis for
terial pathway abundance (PWY.6285..superpathway.of.fatty.acids.biosynthesis..E..coli.) || id:ebi−a−GCST90027577' on 'Pyogenic arthritis || id:
